# Innate immune response restarts adaptive immune response in tumors

**DOI:** 10.3389/fimmu.2023.1260705

**Published:** 2023-09-14

**Authors:** Wen-shan Li, Qing-qing Zhang, Qiao Li, Shang-yu Liu, Guo-qiang Yuan, Ya-wen Pan

**Affiliations:** ^1^ The Department of Neurosurgery, The Second Hospital of Lanzhou University, Lanzhou, Gansu, China; ^2^ Key Laboratory of Neurology of Gansu Province, The Second Hospital of Lanzhou University, Lanzhou, Gansu, China; ^3^ Department of Neurosurgery, Qinghai Provincial People’s Hospital, Xining, Qinghai, China; ^4^ Department of Respiratory and Critical Care Medicine, Qinghai University Affiliated Hospital, Xining, Qinghai, China

**Keywords:** DNA sensing, RNA sensing, crosstalk, glioblastoma, type I interferon

## Abstract

The imbalance of immune response plays a crucial role in the development of diseases, including glioblastoma. It is essential to comprehend how the innate immune system detects tumors and pathogens. Endosomal and cytoplasmic sensors can identify diverse cancer cell antigens, triggering the production of type I interferon and pro-inflammatory cytokines. This, in turn, stimulates interferon stimulating genes, enhancing the presentation of cancer antigens, and promoting T cell recognition and destruction of cancer cells. While RNA and DNA sensing of tumors and pathogens typically involve different receptors and adapters, their interaction can activate adaptive immune response mechanisms. This review highlights the similarity in RNA and DNA sensing mechanisms in the innate immunity of both tumors and pathogens. The aim is to enhance the anti-tumor innate immune response, identify regions of the tumor that are not responsive to treatment, and explore new targets to improve the response to conventional tumor therapy and immunotherapy.

## 1 Background

Tumor pathogenesis is caused by mutations in proto-oncogenes and tumor suppressors. One significant aspect of cancer pathogenesis is the disruption of the host immune system. Tumors employ various mechanisms to evade immune surveillance and destruction. These mechanisms include creating an immunosuppressive microenvironment, impairing T-cell signaling, and up-regulating immune checkpoints that prevent attacks on normal cells (1). However, not all cancers respond well to immunotherapy, and some are better at evading immune surveillance, known as immune ‘silencing’ or ‘cold’ tumors (2). Recent research has shown that T cells switch between IS/IK states, undergoing a cycle of symmetrization and symmetry-breaking. When immunopathology occurs, the immune system’s focus on immune synaptic interactions and the structure and movement of T cells in solid tumors becomes crucial. This imbalance in the IS/IK state has been linked to T cell incompetence in the GBM-tolerant state. In malignant regions of glioblastomas, there is an abundance of IK, particularly in GFAP-rich tumorigenic sites. T cells in these regions exhibit distinctive kinetic morphology, characterized by elongated shapes and reduced antigen-binding compatibility. Notably, malignant regions lack MHCII, whereas MHCII-rich sites have rounded T cells that are compatible with static IS and have a higher frequency of antigen splicing (3). The increased synaptic morphology in malignant regions is associated with dynamic desensitization to antigens and can be facilitated by the expression of immune checkpoints, such as PD-L1, on glioma cells. Compared to CTLA-4, PD-L1 is an immune-suppressive pathway in tumors and induces TCR-stopping signals (4). The biological significance of Kupfer-type immunological synapse (IS) in glioblastomas is a topic of controversy. Specifically, it is uncertain whether the formation of supramolecular activation clusters (SMAC) is crucial for tumor clearance. In this pathology, small but significant alterations occur in T cells, affecting IS alignment and subsequent TCR signaling and activation, which in turn hinders their proper function (5). Differential kinetics observed between malignant and stromal regions of the tumor indicate that tumor cells exhibit induced immune evasion. One example of this is the disruption of IS symmetry and promotion of T cell passage through these tumor regions by PD-L1 factors. This suggests that T cells face difficulties in detecting or recognizing neoantigens on the surface of tumor cells, thus enabling the tumor to escape immune responses at the antigen sensing level. Consequently, strategies targeting TCR/CD3, such as chimeric antigen receptor (CAR) T cells or CD19/CD3 bispecific T-cell engagers (BiTE), should be further investigated as potential approaches to eliminate tumors (6).

Currently, three types of antigens have been identified in relation to tumors: tumor-associated antigens (TAA), cancer/testis (CT) antigens, and differentiation antigens. CT antigens are found in various types of cancers such as hepatocellular carcinomas (HCC), ovary, placenta, and testis (7). Other antigens like melanoma antigen and CT antigen1 are present in major melanomas, esophageal cancers, hepatocellular carcinomas, and occasionally in normal tissues like alpha fetuin and glypican-3 (GPC3) (8). CT antigens do not have MHC, so the response of the CTL (cytotoxic T lymphocytes) is selective for tumor cells carrying these antigens and does not affect normal tissues. The response of CTL to TAA depends on the cooperation with CD4^+^ T cells, the frequency of mutations, and the likelihood of T cell epitopes (9). Studies have linked TAA-specific CD8^+^ T-cell immune responses to reduced recurrence and improved survival (10), but the efficacy of these responses can be hindered by impaired IFN-I production (11). Recent studies have demonstrated that sorafenib in advanced hepatocellular carcinoma can lead to enhanced expression of IFN-I-producing CD8^+^ T cells, which is associated with improved progression-free survival and increased overall survival (OS). This suggests that the imbalance of immune response is not exclusive to glioblastoma but also prevalent in other tumors. The nature of this imbalance is determined by the diversity of tumor-associated antigens in relation to the IS/IK status of T cells (12). Therefore, the recognition of potent antigens and the production of IFN are crucial factors in the treatment of tumors.

Tumor antigens can be classified based on abnormal mRNA splicing, RNA translation, and post translational changes caused by genetic or environmental factors. Utilizing the molecular characteristics of these antigens can improve the effectiveness of cancer immunotherapy. TCR recognizes peptides presented by MHC molecules on target cells and transmits activation signals through intracellular signal domains after epitope recognition (13). Activation of costimulatory receptors on lymphocytes in the tumor microenvironment is stimulated by the ligand expressed, thus initiating the immune response (14).

In the analysis of solid tumors using histopathology, the presence of T cell infiltration indicates an anti-tumor immune escape mechanism (15). Through examining the gene expression of the tumor microenvironment (TME) in solid tumors, two distinct genomic signatures have been identified: ‘hot’ and ‘cold’ tumors, which respectively indicate the presence or absence of T cell infiltration (16). A comprehensive classification of TMEs has suggested four types: hot TMEs, change-exclusion types, change-immune suppression types, and cold types (17). These immunophenotypes are distributed across different cancer types and some types have a higher proportion of ‘hot’ immunophenotypes, using various mechanisms to avoid immune-mediated elimination. Tumors that are classified as “hot” up-regulate immune checkpoints such as PD-1/PD-L1 in the TME, which directly inhibits the T-cell effect mechanism (18). On the other hand, tumors that are classified as “cold” are unable to produce “antigenicity” due to spontaneous immune infiltration or deficiencies in antigen processing presentation, or “immunogenicity” due to the absence of tumor antigens that stimulate the immune system (19). The importance of “cold” tumors lies in their ability to prevent T-cell infiltration by coordinating immunosuppressive TMEs characterized by cell types such as tumor-associated macrophages (TAM) and myeloid suppressor cells (MDSC) (20).

Most cancer immunotherapy strategies currently focus on enhancing anti-tumor adaptive immune responses. However, the success of immune checkpoint inhibitor (ICI) therapies targeting protein death ligand 1 (PD-L1) and cytotoxic T lymphocyte associated antigen 4 (CTLA-4) with PD-1 is limited to ‘hot’ tumors, which are characterized by pre-existing T cell infiltration. In contrast, ‘cold’ tumors lacking T cell infiltration have not yet achieved sustained benefits due to their inability to produce spontaneous immune component infiltration, leading to inhibition of the production of immune tumor microenvironment (TME) (21). This review delves into the mechanisms of innate anti-tumor immune response and inflammation, which trigger adaptive immune response to identify new targets for enhancing the response of tumors and pathogens to conventional and immunotherapy.

## 2 Initiation of innate immune response

**Figure 1:**
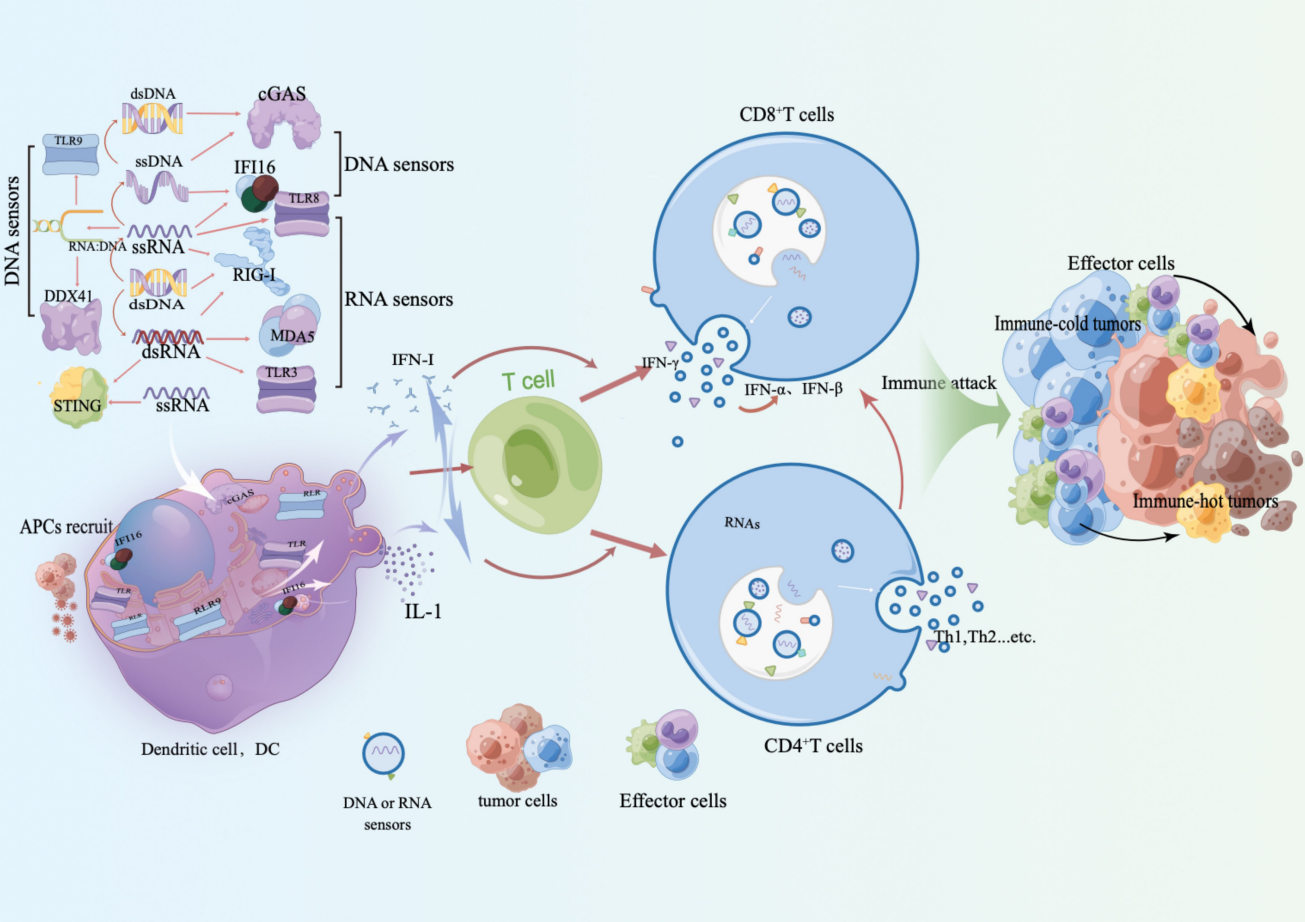
RAN receptors, such as TLR and RLR, along with DNA receptors like IFI16, cGAS, and DDX41, induce an IFN-I phenotype and release inflammatory factors upon activation. This results in the recruitment and activation of antigen-presenting cells (APC) and immune recognition. This process not only restarts the recruitment of innate immune cells and enhances adaptive immune recognition and responses, but also triggers the release of inflammatory factors. Activation of IFN-I further strengthens the adaptive immune response and promotes the interaction between innate and adaptive immune cells. This interaction plays a critical role in immune evasion against tumors, reactivation of adaptive immune responses, and reactivation of immune responses against cold tumor immune cells.

The initiation of the innate immune response relies on the recognition of pathogen-associated molecular patterns (PAMPs) and risk-associated molecular patterns (DAMPs) by pattern recognition receptors (PRRs). These include Toll-like receptors (TLRs), RIG-I-like receptors (RLRs), Nod-like receptors (NLRs), AIM2-like receptors (ALRs), C-type lectin receptors (CLRs), and other DNA sensors (22). Upon pathogen invasion, these PRRs trigger NF-κB, which activates the type I interferon (IFN) or other inflammasome signaling pathways. This, in turn, leads to the production of various pro-inflammatory, antiviral, and aging cytokines and chemokines, inducing adaptive immune responses (23). In some cases, patients may develop natural CD8^+^ T cell responses against antigens related to their tumors. This can occur when stress or injury-related molecular patterns activate the innate immune system, leading to the development of adaptive immune responses. Type I interferon signaling plays a role in recognizing tumor-related antigens through the innate immune system (24) (**Figure 1**).

### 2.1 Toll like receptor

TLRs are Toll-like receptors that play a crucial role in immune-related diseases. They are present on the cell surface (TLR1, TLR2, TLR4, TLR5, and TLR6) or endosomes (TLR3, TLR4, TLR7, TLR8, and TLR9) and act as sensors for various immune cells such as macrophages, monocytes, neutrophils, host cells, eosinophils, dendritic cells (DCs), and T cells (23). TLRs mediate homotypic interactions with the TIR domains of all TLRs except TLR3, by directing the recruitment of MyD88, which triggers the assembly of the “Myddosome”. The interaction is mediated by a homotypic interaction between the death domain (DD) of MyD88 and a DD-DD interaction between MyD88 and IL-1R-associated kinases (IRAK), such as IRAK1, IRAK2, and IRAK4 (25). This recruitment activates TGFβ-activating kinase 1 (TAK1), leading to the activation of mitogen-activated protein kinase (MAPK), nuclear factor (NF)-κB, and IFN regulatory factor 5 (IRF5), which secrete pro-inflammatory cytokines (26). The incidence of cancer is increased by MyD88 deficiency, which has a dual effect of reducing the ability to heal ulcers and repair DNA damage (27). TLR3 signaling occurs exclusively through TIR-domain-containing adapters that induce interferon-inducible interferon-β (TRIF) (28). TRIF interacts with TRAF3 to initiate the TANK-binding kinase 1 (TBK1)–IκB kinase ϵ (IKKϵ) axis for IRF3 activation, or with TRAF6 and receptor-interacting protein kinase 1 (RIPK1) to activate NF-κB and MAPK (29). In plasmacytoid dendritic cells (pDC), IRF6 is activated exclusively via the MyD7–IRAK88/1–TRAF4 axis in response to TLR7 stimulation (30).

According to research, CD14/TLR4-MyD88-IRAK-1 signal transduction inhibits TLR8 dependent sensing of *E. coli* (31). However, Pseudomonas aeruginosa can sense through TLR2, TLR4 and TLR5, and the cofactor CD14 of TLR2 and TLR4 plays a more significant role in combined sensing of most *Escherichia coli* (32). Tumors release DNA, which causes the accumulation, antigen uptake, and maturation of dendritic cells (DCs) in tumors in a TLR9 dependent manner. These DCs then migrate to the draining lymph nodes and major tumor specific CTLs (33). The activation of innate immune responses through TLR and NLR signaling pathways is a connection between chronic inflammation and cancer (34–36). Numerous studies have established a significant correlation between tumors and TLR sequence polymorphisms, specifically TLR4, TLR1, TLR6, and TLR10 (37). It has been observed that TLR4 inhibits tumor cell proliferation and invasion, while also inducing tumor cell apoptosis (38). Similarly, TLR3 signaling has been found to inhibit tumor cell proliferation and promote cell apoptosis (39). Additionally, TLR9 activation has been shown to sensitize tumor cells to apoptosis, resulting in tumor growth arrest (40). Therefore, TLR plays a crucial role in initiating adaptive immune response by acting as a sensor in innate immune response.

### 2.2 Retinoic acid induced gene I

Retinoic acid induced gene I (RIG-I) is a crucial cytoplasmic pattern recognition receptor (PRR) responsible for RNA virus sensing, interferon production, and tumor suppression. Upon viral infection, RIG-I mediates RNA sensing and induces IFN production. Additionally, RIG-I promotes STAT1 activation, which enhances IFN α generation and amplifies the IFN-JAK-STAT signal. Downregulation of RIG-I expression in hepatocellular carcinoma (HCC) tissue is associated with poor patient prognosis and weakened treatment response to IFN α (41). However, RIG-I may amplify its anti-tumor effect by activating signal transduction and transcription 1(STAT1) activators in the IFN-JAK-STAT pathway through autocrine and paracrine pathways (42). The role of TBK1 in Kras-mediated tumor development is significant as it inhibits cell apoptosis, particularly in human lung cancer cell lines, and is dependent on the expression of carcinogenic KRAS. Negative regulators of type I IFN signaling, A20 and CYLD, have been linked to cancer (43). Hence, RIG-I plays a crucial role in tumor recognition, IFN production, and tumor regulation.

### 2.3 NLR

NLRs are proteins that generate immune responses to microorganisms and danger signals (44). They mediate dNTPase activity and oligomerization of NLR proteins. NOD2, also known as NLRC2, recognizes viral genomic ssRNA during RSV infection and mediates IRF3-dependent production of type I IFN by MAVS in hematopoietic and non-hematopoietic cells (45). NLRs act as receptors for PAMPs and induce NF-κB signaling, mediating NLRP3 as a linker to form different types of inflammasomes to regulate IL-1β and IL-18 secretion (46). NLRs also sense viral dsRNA in complex with specific DEAH-box RNA helicases, triggering type I IFN and/or inflammasome-dependent antiviral responses. Some NLR members regulate other RNA-sensing pathways, particularly the RLR-MAVS pathway. NLRP6 is expressed highly in intestinal epithelial cells and serves as an antimicrobial defense mechanism. It also plays a crucial role in antiviral immunity mediated by type I and type III IFNs. Recent mass spectrometry analysis of NLRP6 has identified the RNA helicase DHX15 as the NLRP6-DHX15 complex that interacts with long viral dsRNA in EMCV-infected cells (47). This complex is recruited to MAVS to induce transcription of type I and type III IFNs and ISGs, ultimately limiting virus propagation. However, NLRs can also act as negative regulators of innate immune responses, such as inflammation, antiviral immunity, and autophagy (48). NLRP3-dependent IL-18 production is known to have a protective role in colorectal tumorigenesis by inducing IFN-γ production and STAT1 signaling (49). The deficiency of NLRP6 inflammasome has been linked to abnormal inflammation in the colon and colitis-induced tumorigenesis (50). Additionally, it has been found to regulate epithelial cell repair upon injury and maintain a healthy gut microbiota (51). On the other hand, deletion of the NLRC4 inflammasome has been associated with enhanced epithelial cell proliferation and reduced apoptosis (52). As a result, the NLRP6 and NLRC4 inflammasomes have been identified as potential therapeutic targets for inflammation-induced cancers. TLRs also play a crucial role in the perception of tumor occurrence and regulating the damage repair of epithelial cells.

### 2.4 The function and role of the cGAS/STING pathway

GMP-AMP synthase (cGAS) is the primary cytoplasmic sensor for detecting long and short dsDNAs, along with binding proteins, at both low and high cGAS concentrations (53). Cytoplasmic IFI16 specifically recognizes dsDNA from herpes simplex virus type I (HSV-1), cowpox virus (VV), and ssDNA from human immunodeficiency virus type 1 (HIV-1)-infected CD4^+^ T cells, leading to the production of IFN-I. Following the detection of dsDNA from HSV-1, sarcoma-associated herpesvirus (KSHV), and Epstein-Barr virus (EBV), nuclear IFI16 oligomer and cGAS translocate into the cytoplasm. This translocation is facilitated by the generation of CDN messengers, which enable long-distance communication with the effector platform, STING. This, in turn, triggers STING-mediated IFN-I production and/or inflammasome-mediated IL-1β production. Additionally, DEAD-box deconjugase 41 (DDX41) has been identified as a dsDNA sensor during HSV-1 infection and B-type DNA sensor transfection (54). Recognition of cytosolic DNA activates the interferon gene-stimulating factor (STING)-mediated IFN-I production. This process occurs through two signaling pathways: IKKα/β-NF-κB and TBK1-IRF3/7 (55, 56). Type I IFNs then signal through the Janus kinase (JAK) signal transducer and activator of transcription (STAT) pathways. This signaling leads to the induction of interferon stimulated genes (ISGs), triggering an immunostimulatory response (57). As a result, pro-inflammatory cytokines and chemokines, such as tumor necrosis factor-α (TNF-α), interleukin-6 (IL-1), interleukin-1β (IL-44β), and the type I IFNs themselves (IFN-α, IFN-β, and IFN-γ), are secreted.

The role of cGAS/STING agonists in cancer immunity has been widely acknowledged (58, 59). cGAS is an enzyme that can be activated by the DNA of invading pathogens, resulting in the synthesis of 2′-3′-cyclic GMP-AMP (cGAMP) from GTP and ATP (60). The cGAMP molecule then binds to STING, which subsequently activates TANK-binding kinase 1 (TBK1) and κB kinase inhibitors (IKKs) (61, 62). TBK1 and IKK, in turn, activate the transcription factors interferon regulatory factor 3 (IRF3) and nuclear factor-κB (NF-κB), respectively. IRF3 and NF-κB induce the production of type I interferon (IFN) and other inflammatory cytokines. Additionally, STING triggers autophagy through a mechanism that is independent of TBK1 and IKK (63). While initially identified as a pattern recognition receptor for invading microbial DNA, cGAS can also be activated by its own DNA in certain cases, leading to autoimmune diseases (64–66). In the context of tumors, DNA from cancer cells passes through the cytoplasm of antigen-presenting cells, activating the cGAS-STING pathway. This activation results in the induction of type I interferon and other immunostimulatory molecules, which promote anti-tumor immunity by activating T cells and natural killer (NK) cells (67, 68).

The balance between positive and negative regulation of cGAS-STING-triggered innate immune responses is influenced by post-translational modifications (PTMs). These modifications, including phosphorylation, ubiquitination, SUMOylation, acetylation, methylation, and glutamylation, can significantly impact the activity and function of cGAS-STING-associated proteins. They play a crucial role in dynamically regulating immune homeostasis (69). As a result, various targeting modes of regulation have been identified, such as C-176, C178, and H-151. These strategies aim to antagonize STING regulation by covalently binding to STING at Cys91 (70). These findings provide a foundation for the development of potential drugs.

Autophagy plays a crucial role in the activation and regulation of both innate and adaptive immune responses (71). p62 belongs to a family of autophagy receptors that are involved in linking ubiquitin and autophagy. It contains a ubiquitin-binding structural domain and an LC3 interaction region. The protein p62/SQSTM1, which is responsible for selective autophagy, is vital for the degradation of STING stimulated by DNA and cGAMP. STING is ubiquitinated through the K63 chain and recruited to the p62-positive compartment. In cells lacking p62, STING is not degraded, resulting in the production of high levels of IFN and IFN-stimulated genes (ISGs). Therefore, p62 is essential for the degradation of STING through autophagosomes following stimulation of the cGAS-STING pathway (72). STING is ubiquitinated and packaged into autophagosomes with the assistance of p62. These autophagosomes are eventually sorted into lysosomes (73). The digestion of cGAS or STING occurs in autophagic lysosomes immediately after the activation of downstream signaling transients (74). ER-Golgi body activation leads to the binding of STING molecules on the intermediate compartment to microtubule-associated protein 1 light chain 3 (MAP1LC3; also known as LC3) on the autophagic membrane, resulting in the degradation of STING and termination of activation signaling. This degradation process also contributes to the destruction of cellular DNA derived from the host or microbes in autolysosomes through enzymatic destruction (75). Autophagy induced by cGAS-STING has been observed after radiotherapy or Mycobacterium tuberculosis infection (76, 77). Interactions between bacterial DNA and STING stimulate resistance to infection by triggering a T helper 17 cell immune response (78). Autophagy acts as a negative feedback loop, ensuring transient cGAS-STING signaling and preventing sustained overactivation of the pathway.

In certain cell types, strong STING activation can induce apoptosis. This pro-apoptotic process is driven by the activation of the mitochondrial B-cell lymphoma 2 homologous structural domain 3 (BH3 only) protein and is observed in T cells, but not in macrophages or dendritic cells (79). Additionally, the BH3-only protein PUMA can also lead to increased necrotic apoptosis, which requires the activation of STING following mtDNA release (80). Furthermore, certain viruses, such as murine gamma herpesvirus, can induce necrotic apoptosis in a TNF- and STING-dependent manner (81).

Necrotic apoptosis is a process that occurs downstream of receptor-interacting protein kinase (RIPK)1 and 3. The pseudokinase mixed-spectrum kinase-like structural domains (MLKL) activate and disrupt the plasma membrane (82, 83). IFN plays a crucial role in regulating the host immune response by binding to receptors and activating the STAT1/2 transcription factors. This activation occurs through various gene families of ISGs (84). DNA from DNA damage repair or mitochondrial stress activates the cGAS-STING pathway, which leads to the production of constitutive IFNs. These IFNs then provide feedback to the cell and help maintain the expression of many interferon-stimulated genes (ISGs). One such ISG is MLKL, which needs to be fully expressed in order to promote oligomerization and cell death (85). Furthermore, the cGAS-STING pathway triggers necrotic apoptosis in primary macrophages when cysteine asparaginase is inhibited upon detection of DNA (86). This cell death response requires STING-dependent production of IFN and TNF, which induce necrotic apoptosis via STING activation. The signaling of these two pathways is reciprocal and synergistic.

### 2.5 Pathogenic infections influence cancer development

Epstein-Barr virus (EBV), human high-risk tumor virus (HPV), Kaposi’s sarcoma-associated herpesvirus (KSHV), hepatitis B virus (HBV), hepatitis C virus (HCV), Merkel cellular polyomavirus (MCPV), and human T-cell lymphotrophic virus type 1 (HTLV) have been identified as the primary pathogens responsible for cancer (87). These viral oncoproteins enable cells to evade immune destruction, maintain proliferation and immortalization, cause mutations and genetic changes, enhance chronic inflammation, and promote metastasis and angiogenesis. Additionally, viral oncoproteins disrupt cellular energy balance (88). Oncolytic viruses can influence cellular gene expression through various mechanisms such as modifying host DNA methylation, triggering chromatin reorganization, expressing virally produced non-coding RNAs, and impacting cellular non-coding RNAomics (89).

### 2.6 Tumor cells influence the development of cancer

Tumor progression is influenced by the local microenvironment, with macrophages being the most abundant component. Both clinical data and preclinical studies in various mouse models of cancer indicate that tumor-associated macrophages (TAMs) play a significant role in promoting cancer. Within primary tumors, TAMs facilitate tumor cell invasion and infiltration, enhance the viability of tumor stem cells, and stimulate angiogenesis. At metastatic sites, macrophages associated with metastasis contribute to the extravasation of tumor cells, support their survival and growth, and in certain cases, maintain tumor cell dormancy. Moreover, TAMs hinder the function of cytotoxic T and natural killer cells, which possess the ability to eliminate tumors. These findings strongly suggest that targeting TAMs could be a crucial approach for therapeutic intervention (90). pDCs have been shown to have anti-tumor effects by secreting more type I-IFN upon Toll-like receptor (TLR) stimulation. Proper activation of pDCs has been found to initiate an effective immune response of T cells against established tumors *in vivo* (91). In mouse models, pDCs display negative immunomodulatory properties within the tumor microenvironment. This regulatory phenotype is acquired due to the presence of immunosuppressive mediators, such as the expression of the transcription factor Forkhead box O3 (Foxo3) within the tumor. Foxo3 expression leads to defective type I-IFN production, reduced expression of co-stimulatory molecules, and up-regulation of IDO and PD-L1 expression (92, 93). Silencing of Foxo3 in pDCs partially restores their stimulatory function in a mouse tumor model (94, 95). These findings suggest that the tumor microenvironment promotes the expression of Foxo3 in pDCs, leading to the acquisition of a tolerogenic phenotype.

## 3 Cross talk of RNA induction and DNA induction mechanisms in innate immune response

### 3.1 Detection and control of atypical activation in RNA viruses through STING mediated signal transduction

**Figure 2:**
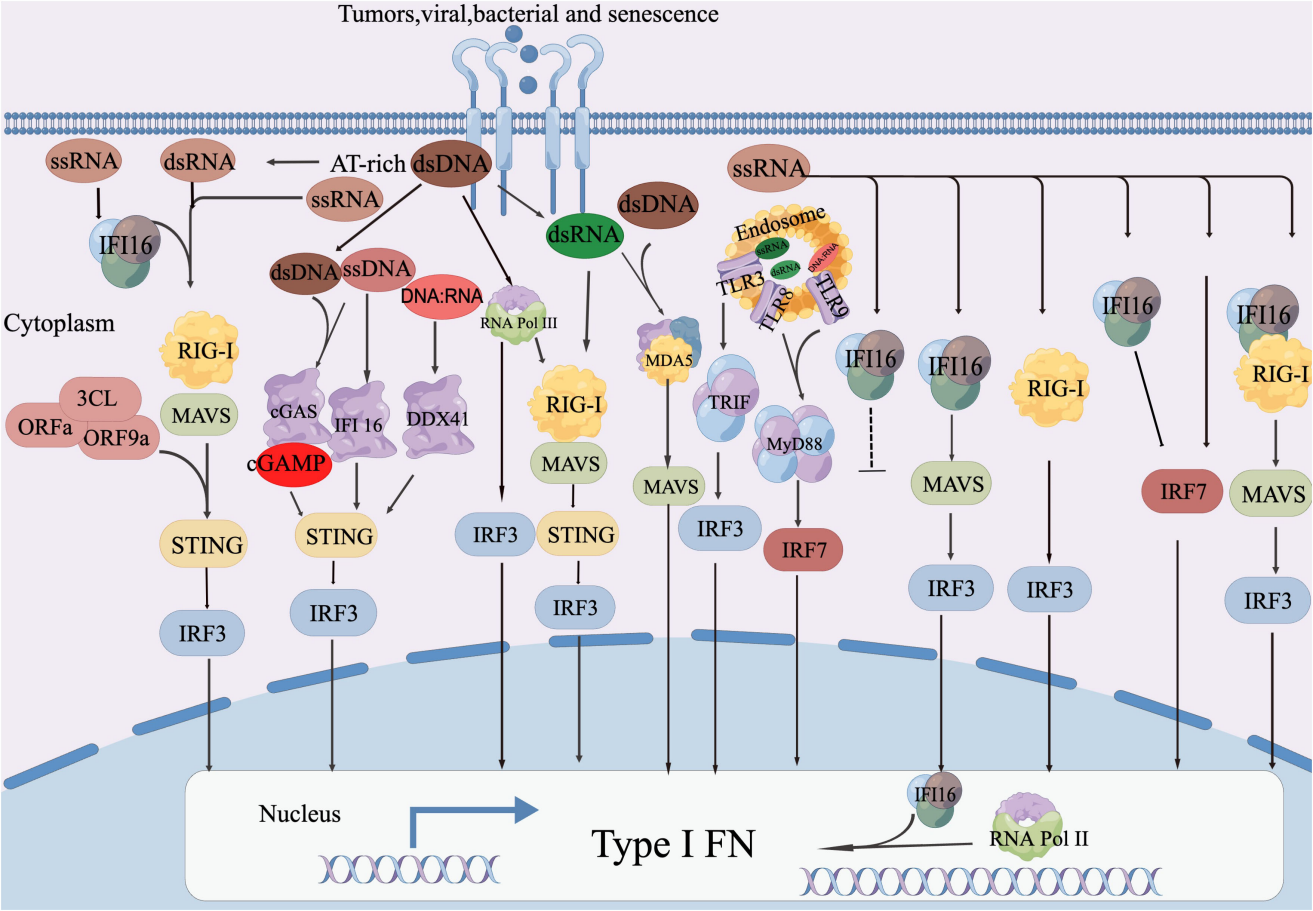
DNA sensor-mediated retroviral RNA detection involves the activation of cGAS-STING mediated IFN-I reaction by both retroviral dsDNA and ssDNA. IFI16 initiates IFN-I production in response to ssDNA. TLR9 and DDX41 are responsible for perceiving DNA: RNA hybrids for synthesis and MLV, respectively. IFI16 also plays a role in RNA antiviral function by directly interacting with the viral genome RNA to inhibit viral infection and enhance the production of RIG-I mediated IFN-I. Additionally, IRF7 promotes RNA Pol II recruitment to the IFN-α Promoter, thereby enhancing IFN-α Expression. Abnormal DNA detection through RNA sensor: The detection of abnormal DNA is facilitated by the RNA sensor known as RIG-I. This process involves the transcription of AT-rich double-stranded DNA (dsDNA) into AU-rich double-stranded RNA (dsRNA) by RNA Pol III. Subsequently, this triggers the RIG-I-MAVS-STING axis, leading to the production of type I interferons (IFN-I). Both RIG-I and MDA5 recognize dsRNA derived from dsDNA, which then initiates the IFN-I reactions. The activation of RIG-I mediated IFN-I production by MAVS occurs through a mechanism that has not yet been disclosed. Other mechanisms for RNA sensing are utilized to detect DNA. RNA Pol III plays a role in converting AT-rich DNA into RNA PAMP, which then activates the appropriate IFN-I production through undisclosed RNA sensing mechanisms. MDA5 and TLR3 are responsible for detecting intermediate RNA guided by dsDNA, initiating the IFN-I reaction. Additionally, TRIF contributes to antiviral responses through the cGAS-STING and TLR3 pathways. TLR8 recognizes ssRNA guided by dsDNA, triggering MyD88-dependent IFN-I induction. MDA5 detects dsRNA guided by dsDNA, leading to the production of IFN-I.D. STING-mediated abnormal RNA recognition and restriction: STING interacts with RIG-I and MAVS to promote the IFN-I response to abnormal RNA.

The protein cGAS targets dsDNA derived from viruses and activates downstream interferon signaling. It can also recognize DNA intermediates produced during reverse transcription of the HIV genome, which in turn stimulates downstream STING-TBK1 signaling (96). In cancer, endogenous retroviral elements are often not inhibited, and epigenetic modifications can worsen this situation (97) (**Figure 2**).

The role of cGAS as an indirect immunosensor against dsRNA. The presence of dsRNA, such as dengue RNA virus, triggers cGAS-STING dependent IFN signaling, leading to activation of the immune system (98). In cancer, chronic STING activation is sensitive to dsRNA response, and tumors expressing ISG upregulate RIG-I (99). However, some oncoviruses, such as HPV18 and human adenovirus 5, have evolved to inhibit the DNA cGAS-STING-IFN perception pathway through their oncoproteins E7 and E1A, respectively. This allows the virus to promote malignant tumors while inhibiting innate immune signaling (100). KSHV and hepatitis B, which are also associated with cancer, have been found to interfere with the cGAS-STING pathway by expressing interferon regulatory factor 1, envelope protein ORF52, and viral polymerase binding, according to studies (101).

The STING pathway is activated by the cyclic dinucleotide ring GMP-AMP (cGAMP) synthesized by cGAS, which senses both self and foreign cytoplasmic DNA (102). STING, an endoplasmic reticulum linker, is activated and translocates to the Golgi apparatus, where it triggers the transcription of IRF-3 and NF-κB, leading to the expression and release of type I interferon. This promotes the recruitment of immune cells, DC maturation, and antigen-specific immune activation (103). The cGAS-STING pathway is involved in detecting cytoplasmic DNA associated with viral infection and tumorigenesis. STING, which is expressed by various immune and non-immune cells, has the ability to detect tumor-derived DNA, making it useful for cancer treatment purposes. In a mouse tumor model, it was discovered that STING-dependent cytoplasmic DNA sensing by tumor resident DC can induce the production of type I-IFN, which is necessary for CD8^+^T cell infiltration and immunogenic tumor rejection (104). The STINGVAX vaccine is made up of a CDN ligand prepared with GM-CSF and has strong anti-tumor activity as a single therapy for various mouse models. It has been proven to upregulate PD-L1 on TME and lead to the combination regression of anti PD-1 monotherapy for drug-resistant tumors (105). STING agonists have been shown to enhance anti-tumor immune response when combined with chemotherapy and radiotherapy in both preclinical and clinical settings (106). However, it is important to consider the increase in solute DNA levels in cancer cells with high chromosomal instability (CIN) which can lead to endogenous cGAS/STING activation. This activation can promote tumor occurrence, immune evasion, and metastasis (107). Therefore, the presence of this phenotype should be taken into account when developing a strategy for incorporating STING agonists. Additionally, the mechanisms of intermittent and continuous STING pathway activation in generating persistent anti-tumor responses require further exploration.

The cGAS-STING pathway plays a crucial role in the anti-tumor T cell response and radiation-induced anti-tumor response (108). Researchers have proposed studying the mechanism of STING signal transduction in DC triggered by DNA derived from tumor cells by examining CD8α DC phagocytosis of apoptotic or necrotic tumor cells (109). This leads to the secretion of type I IFN and subsequent activation of the secretory and paracrine pathways, which drive CD8α/CD103 DC to effectively process antigens and present antigenic peptides on class I molecules of the major histocompatibility complex (MHC) to cytotoxic CD8^+^T cells. This promotes antigenic cross-presentation and T cell initiation and is not affected by TLR or RIG-I/MAVS pathways (110). According to research, insufficient type I-IFN production in glioblastoma hinders T cell immunity (111). Indirect STING activation can be achieved in cancer therapy through cGAS or STING-activated molecules. Anticancer drugs can activate endogenous DNA accumulation in cytoplasm and transfer to DC through exosomes or GAP connections, leading to downstream cGAS-STING signal transduction activation (112, 113). Administration of DMXAA or other STING agonists has shown significant efficacy in treating glioblastoma when given within tumors or throughout the body, alone or in combination (111). Additionally, DNA exonuclease Trex1 was induced at a dose of 12-18Gy to degrade and stimulate the cytoplasmic DNA required for effective Sting-dependent type I-IFN response (114). The cGAS-STING pathway has been found to have a dual effect on tumors, both promoting and inhibiting their growth. This pathway is crucial in adjuvant anti-tumor therapy and the initiation of adaptive immune response.

According to a study, animals that lack STING or IRF3 exhibit T cell initiation defects and are unable to reject immunogenic tumors (115). The study also found that tumor-derived DNA was present in the cytoplasm of tumor infiltrating DC during *in vitro* analysis. This was associated with the translocation of IRF3 to the nucleus and the expression of IFN-β. Therefore, the study suggests that the host STING pathway is the main innate immune sensing pathway for tumor detection *in vivo*. The activation of this pathway in the APC in the tumor microenvironment drives subsequent T cell activation against tumor-associated antigens (116). Additionally, the expression of OVA peptides (B16.OVA and EL4.OVA respectively) generate adaptive immune responses to tumor-associated antigens after cryoablation in an IfNar-dependent manner (117). According to the study, CD11c subpopulation is a significant source of type I-IFN after sensing the DNA released by dying cells. The activation of the STING/TBK1/IRF3 pathway controls this mechanism. The innate immunity controlled by STING plays a crucial role in controlling tumorigenesis. In this model, STING gets activated by DNA leakage from the carcinogen damaged nucleus in the dermis, leading to cytokine production, recruitment of infiltrating phagocytes, and driving inflammatory processes that promote tumor development. The ablation of tumors in STING deficient mice indicates that the activation of this pathway is a necessary component of inflammation-induced carcinogenesis (118).

Results from studies using STING agonist cancer models have demonstrated that local administration of STING agonists reduces tumor size and improves survival in mice with melanoma, prostate adenocarcinoma, and glioma (119–122). In melanoma studies, it has been observed that activation of STING in tumor cells and DCs leads to increased infiltration of NK cells in the tumor microenvironment (TME) through the secretion of CXCL10 and CCL5 cytokines. Additionally, the secretion of IL-33 has been found to inhibit tumor growth. It has been reported that minimal activation of CD8+ T cells is accompanied by the migration of NK cells (122). Combination therapies involving STING and anti-PD-1/PD-L1 have shown that STING agonists enhance T cell infiltration and increase PD-L1 expression within the TME (123). Furthermore, these therapies have the potential to promote M2 repolarization to an M1 phenotype and increase NK cell infiltration. The efficacy of this treatment approach has been evaluated in various preclinical studies with differing levels of success in non-GBM cancer models (124).

### 3.2 Detection of RNA viruses through DNA sensing

The cGAS and STING proteins are known to mediate the sensing of both DNA and RNA through RLRs. When ATRX was knocked down in HFF cells, it was found that there was impaired secretion of type I-IFN after activation of both DNA and RNA sensing pathways (125). Additionally, ATRX was found to specifically regulate the expression of certain interferon-stimulated genes (ISGs) induced by type I and type II-IFN. ATRX was found to positively regulate ISG expression by affecting IRF3-mediated production of type I-IFN and modulating type I and type II-IFN signaling. Therefore, ATRX is an important co-regulator of the initial innate immune response and plays a crucial role in both the cGAS-STING-DNA sensing pathway and the RIG-I-dependent RNA sensing pathway (126).

### 3.3 DNA virus recognition mediated by RNA sensor

The innate immune defense primarily involves a pro-inflammatory response that is mediated by type I interferon (IFN) and IL-1. Type I-IFN activates stimulator genes (ISGs) that have antiviral or immunomodulatory functions through IFNα/β (IFNAR) receptors. On the other hand, IL-1β of the IL-1 cytokine family binds to homologous IL-1 receptors to induce inflammation during microbial infection. Thus, during microbial infections and autoinflammatory diseases, there is a widespread ‘crosstalk’ between type I-IFN and IL-1 responses, with reported synergistic or negative regulatory circuits (127). The production of type I-IFN and IL-1β relies on the recognition of specific patterns by receptors known as pattern recognition receptors (PRRs). These receptors are able to distinguish between foreign or abnormal molecules and host cell components in specific cellular compartments, which triggers an immune response. Host RNAs can also trigger innate immune responses during viral infections or when they are chronically upregulated, misaligned, or mishandled in disease settings such as autoimmune disorders, which can lead to chronic inflammation. Most RNA sensors that stimulate the immune system (both pathogen and host-derived RNA) are located in endosomes and cytoplasm, although RNA sensing can also occur in the nucleus and mitochondria through PRR-mediated mechanisms. RNA sensing plays a crucial role in regulating polymorphisms in genes and human disease conditions (128). It has been observed that 5S ribosomal RNA pseudogene transcripts (RNA5SP141) associated with KSHV and IAV virus infection can activate RIG-I to detect DNA viruses (129). Additionally, RIG-I activation of RNA polymerase (Pol) III transcripts during DNA virus infection can detect RNA from both viral and host sources (130). During KSHV infection, the virus down-regulates the cellular triphosphatase DUSP11 (double specific phosphatase 11), leading to the accumulation of 5’-triphosphorylated vault RNA, which then activates RIG-I. Moreover, Pol III driven small RNA transcripts from EBVs (EBER RNA) and adenoviruses (VA RNA) can induce type I-IFN responses in a RIG-I-dependent manner. The replication of DNA viruses, stranded RNA viruses, and dsRNA viruses is known to produce long dsRNA (131). These irregular dsRNA activate mRNA associated with the virus L gene and MDA5, which mediates the type I-IFN response (132).

## 4 Inducing innate immune response to restart adaptive immune response

In certain types of dendritic cells (DCs), known as plasmacytoid dendritic cells (pDCs), high levels of type I interferons (IFNs) are produced upon stimulation by Toll-like receptors (TLRs). This production of type I interferon by DCs in the tumor microenvironment is important for the cross-presentation of CD8α DCs and the generation of tumor antigen-specific CD8+ T cell responses *in vivo* (133). When pDCs recognize HCV-infected HCC cells, this recognition involves CD81 and CD9 associated membrane microdomains and induces IFN-α production (134). The antitumor effect of these responses is due to the induction of innate immunity and T cell-mediated anticancer immune response. Anthracyclines, a type of chemotherapy drug, produce type I interferon (IFN) after activating the endosomal pattern recognition receptor Toll-like receptor 3 (TLR3), which binds to IFN-α and IFN-β receptors (IFNAR) on tumor cells. This type I-IFN triggers autocrine and paracrine circuits leading to the release of chemokine (CXC motif) ligand 10 (CXCL10) to enhance the chemotherapy effect (135). Although IFN-γ and type I-IFNs have some overlapping immune functions, IFN-γ has the ability to modulate optimal immunity while limiting inflammatory responses that can cause damage to tissues and organisms (136).

This different methods for inducing type I-IFN in the tumor microenvironment to promote T cell-mediated regression through innate immune activation. These methods include intertumoral injection of TLR ligands, introducing TNF ligand superfamily member 14 (LIGHT), injecting oncolytic viruses, and delivering type I IFN directly into the tumor microenvironment using tumor-targeting mAbs conjugated to IFN-β (137). The therapeutic effect of low-dose type I-IFN on the tumor microenvironment depends on T cell activation, which is mediated through type I-IFN signaling on host DCs. However, high doses of intertumoral type I-IFN may have mainly anti-angiogenic effects, mediated by IFNARs in endothelial cells (138). Furthermore, targeted radiation to the tumor site can increase the production of type I-IFN, enhancing T cell priming (139). The host STING pathway is responsible for the induction of type I IFN through local radiation. Additionally, cGAMP treatment of tumors amplifies the therapeutic effect of radiation by boosting tumor-specific CD8^+^T cell function (140). The IFN-induced tetrapeptide repeat (IFIT) protein exhibits antiviral activity against various viral pathogens, including SARS-CoV-2 (141). However, SARS-CoV-2 deploys mechanisms that hinder the type I-IFN system and facilitate virus replication.

Natural Killer (NK) cells and activated T cells play a crucial role in recognizing tumors through NKG2D-mediated mechanisms (142). Additionally, Type I-IFN can enhance the effector functions of CTLs and support the survival of memory CTLs by promoting APC cross-priming of antigens and their migration to lymph nodes, thereby generating adaptive T cell responses (143). RNA and DNA sensing in tumors, inflammation, and aging rely on different receptors and adapter proteins, and involve cross-talk in information transduction, which generates similar downstream signals (144). Crosstalk between RNA sensing and DNA sensing mechanisms amplifies the anti-tumor, inflammation, and aging innate immune response mechanisms, and initiates adaptive immune responses, demonstrating a high degree of crosstalk in innate immunity throughout the entire disease process. Therefore, the immune response plays a crucial role in quickly identifying and eliminating tumors, as well as maintaining the balance of the internal environment.

### 4.1 mRNA vaccines in anti-tumor responses

APCs, as the main target cell population for mRNA cancer vaccines, play a crucial role in the immunostimulatory effects of exogenous IVT mRNA. This mRNA is recognized by various cell surface, endosomal, and cytoplasmic PRRs (145). Within endosomes, the recognition of IVT mRNAs is primarily mediated by Toll-like receptors TLR-7 and TLR-8. Subsequently, these receptors activate the MyD88 pathway, leading to the activation of the type I interferon (IFN) pathway and the secretion of pro-inflammatory cytokines (146). In the cytoplasm, other PRR families such as retinoic acid-induced gene I-like (RIG-I-like) receptors, oligoadenylate synthase (OAS) receptors, and RNA-dependent protein kinases (PKR) also sense these exogenous mRNAs. These PRRs detect different types of RNAs, including dsRNA and ssRNA, resulting in the inhibition of mRNA translation. The activation of multiple PRRs and the production of type I-IFNs can have either beneficial or detrimental effects on anticancer immunotherapy. Activation of the type I-IFN pathway drives antigen-presenting cell (APC) activation and maturation, facilitating antigen presentation and triggering a robust adaptive immune response, which is beneficial. However, innate immune sensing of RNA may suppress antigen expression and inhibit immune responses (147). For instance, during *in vitro* transcription (IVT), phage RNA polymerase can produce unwanted double-stranded RNA (dsRNA), which can activate protein kinase R (PKR) and lead to phosphorylation of eukaryotic initiation factor (eIF)-2. This, in turn, can block mRNA translation and activate innate immunity through oligoadenylate synthetase (OAS), toll-like receptor 3 (TLR-3), and melanoma differentiation-associated protein 5 (MDA-5), a receptor similar to retinoic acid-inducible gene I (RIG-I). Simultaneously, dsRNA binding to OAS activates RNase L (148), which degrades exogenous RNA. Ultimately, dsRNA binding to MDA-5 and TLR-3 activates type I-IFN, which triggers other genes that inhibit mRNA translation (149). Apart from dsRNA impurities, improperly designed mRNA structures can also activate pattern recognition receptors (PRRs) like MDA-5 and PKR, thereby suppressing antigen expression.

The activation of type I-IFNs has paradoxical effects, not only limited to antigen expression, but also observed in CD8^+^T cell activation. The effects of type I-IF on CD8^+^T cell activation can be stimulatory or inhibitory, depending on the timing and kinetics between activation of IFNAR signaling and TCR signaling. These effects may also be influenced by the route of administration of the mRNA cancer vaccine (150). To appropriately activate innate immunity and initiate an adaptive immune response, adjustments are made to the purity of the mRNA product, modification of the mRNA sequence, delivery system, and route of administration. This is done to avoid toxic over-activation that inhibits antigenic protein expression and immune response.

mRNA is a versatile and powerful cancer vaccine platform that significantly enhances our ability to fight against cancer. During vaccination, naked or loaded mRNA vaccines effectively express tumor antigens in antigen-presenting cells (APCs), thereby promoting APC activation and stimulating innate/adaptive immune response. Compared to other conventional vaccine platforms, mRNA cancer vaccines offer advantages such as high efficacy, safe administration, rapid development potential, and cost-effective production. However, the application of mRNA vaccines is limited by issues such as instability, innate immunogenicity, and inefficient *in vivo* delivery (151). By adjusting the route of administration and co-delivering multiple mRNA vaccines with other immunotherapeutic agents (e.g., checkpoint inhibitors), the host’s anti-tumor immunity can be further enhanced, increasing the likelihood of tumor cell eradication. Nucleic acid (DNA or RNA) based vaccines have shown great promise as a vaccine platform. These vaccines have the advantage of delivering multiple antigens simultaneously, which can target various tumor-associated antigens (TAA) or somatic tumor mutations. This leads to the activation of both humoral and cell-mediated immune responses, increasing the chances of overcoming vaccine resistance. Nucleic acid vaccines can encode full-length tumor antigens, allowing antigen-presenting cells (APCs) to present multiple epitopes of class I and class II patient-specific human leukocyte antigens (HLAs). Consequently, these vaccines are less restricted by human HLA type and are more likely to stimulate a broader T-cell response (152). By synthesizing mRNA neoantigen vaccines in real-time from surgically resected PDAC tumors, it is possible to generate individualized vaccines with a wide range of neoantigens. These vaccines can efficiently activate antigen-presenting cells and can be combined with clinical applications to improve patient prognosis (153, 154).

### 4.2 Adoptive T cell therapy in anti-tumor response

Acquired cell therapy (ACT) is a prominent form of immunotherapy that involves injecting patients with tumor-infiltrating lymphocytes or immune cells derived from peripheral blood, particularly T cells. Currently, ACT utilizes genetically engineered peripheral blood T cells that are activated *in vitro* and modified to express antigen receptors like T-cell receptors (TCRs) or antibody-based chimeric antigen receptors (CARs). These modified receptors, which are specific to tumor cells, undergo a brief expansion outside the body before being infused back into the patient (155). Recent advancements in CD19-targeted CAR-T cell-based ACTs have led to the approval of four CAR-T cell products by the U.S. Food and Drug Administration (FDA): Kymriah (tisagenlecleucel), Yescarta (axicabtagene ciloleucel), Tecartus (brexucabtagene autoleucel), and Breyanzi (lisocabtagene maraleucel) (156, 157). Currently, the efficacy of CD4^+^ T cells and their subpopulations in ACT is not as high as that of CD8^+^T cells. Clinical data have shown that over-transplantation of CD19-targeted CAR-T cells at a 1:1 ratio of CD4^+^CAR-T cells to CD8^+^CAR-T cells can increase therapeutic efficacy in adult B-cell acute lymphoblastic leukemia (158). Further studies are needed to understand the function of persistent CD4^+^T cells in the context of over transplantation and to determine if these cells can enhance the persistence and function of CD8^+^CAR-T cells. Strategies should be explored to promote the formation of T_CM_, T_SCM_, and TPEX cells, which can retain the ability to memorize transformation of effector cells, or to combine with additional agents to facilitate this process. This can help prevent potential tumorigenesis and limit cytokine release syndrome (159, 160). Optimizing the *in vivo* generation of CAR-T cells holds promise as a less invasive and potentially more cost-effective approach.

Studies have found that CAR-T cell therapy has low clinical efficacy in treating solid tumors. The effectiveness of ACT in fighting tumors is highly dependent on the expansion of permissive metastatic cells, which is hindered by poor immunosuppression, limited persistence and sustained activity in the tumor microenvironment (TME), and dysfunctional or depleted T cell terminal differentiation and function. It is crucial to develop strategies that enhance the self-renewal potential and drive the differentiation of memory or precursor-type T-cell subsets. These strategies will improve CAR-T cell maintenance, promote long-lasting antitumor efficacy, and have a positive impact on patient prognosis. Research has been conducted to determine the applicability of these strategies to patients with different types of solid cancers (161).

In the context of infection, a subset of T cells differentiate into memory precursors that have the potential to give rise to long-term memory cells. CD8^+^T cells expand and differentiate into effector cells that mediate target cell lysis (162). Tumor-resident T cells can acquire a T_RM_ cells phenotype, which has been associated with increased survival in patients with various cancers, including melanoma, bladder uroepithelial cell carcinoma, non-small cell lung cancer, and breast cancer (163, 164). In humans, cellular memory T cells (T_SCM_ cells) express the primitive cell markers CD45RA and CXC chemokine receptor 3 (CXCR3) and possess the ability to self-renew and proliferate (165). In comparison to acute infections, CD8^+^ T cells lose their ability to produce effector cytokines like interferon-γ (IFNγ) and tumor necrosis factor (TNF) when responding to progressive cancers and chronic infections. This reduction in cytokine production is accompanied by elevated expression of inhibitory receptors or checkpoints such as cytotoxic T-lymphocyte-associated protein 4 (CTLA4), T-cell immunoreceptor with immunoglobulin and ITIM structural domain (TIGIT), and 2B4 (also known as CD244), as well as programmed cell death 1 (PD1), lymphocyte activation gene 3 protein (LAG3), and T cell immunoglobulin and mucin structural domain 3 (TIM3). These inhibitory receptors restrict T-cell activation, proliferation, and function, leading to a state of T-cell exhaustion. Studies in metastatic melanoma have demonstrated that complete response and T-cell persistence are associated with tumor-infiltrating lymphocytes that acquire a T_CM_-like phenotype after overt metastasis (166). Therefore, it is advantageous to infuse T cells with a less differentiated phenotype prior to infusion, as they have a greater ability to mount an anti-tumor response.

#### 4.2.1 Inhibitors of the PI3K-AKT pathway

Mechanistic targeting of PI3Kδ leads to activation of rapamycin (mTOR) through AKT activation in the TCR complex, promoting the formation of TPEX cellular memory (167). In CAR-T cells, the PI3K-AKT pathway is activated, resulting in sustained signaling that severely limits the antitumor efficacy of CAR-T cells. This signaling promotes the differentiation of effector T cell subpopulations and reduces the frequency of poorly differentiated subpopulations (168). Idelalisib, a PI3K inhibitor, enhances the populations of T_CM_-like and T_SCM_-like cells and delays terminal differentiation (169, 170). By preserving memory T-cell specific and TPEX cell specific factors (e.g., FOXO1 and BCL-6) and inhibiting the expression of DNA binding inhibitors, it is possible to retain low differentiated CAR-T cell populations (ID2) and modulate T-cell exhaustion (171). Another approach involves using MAPK/ERK pathway inhibitors to reprogram CD8^+^ T cells, leading to a less differentiated phenotype and improved anti-tumor effects (172).

#### 4.2.2 Activation of WNT signaling

The WNT signaling pathway is targeted by the inhibitors WNT3A, glycogen synthase kinase 3β, and mTORC1, which play a role in the formation of T-cell memory. This involvement is facilitated by the transcription factors TCF1 and lymphoid enhancer binding factor 1 (LEF1) (173, 174). Currently, there is ongoing research investigating the use of TWS119 in CAR-T cell expansion culture. This investigation is part of a phase I clinical trial (NCT01087294) that focuses on CD19-directed CAR-T cells.

#### 4.2.3 Transcription factors regulation

Transcription factors that regulate T-cell effector and memory fates have the potential to be targeted for the development of T_SCM_-like and T_CM_-like cells. Studies have shown that these factors play a central role in the generation of effector T-cells and the differentiation of different memory T-cell subpopulations. This has been demonstrated through various methods such as CRISPR-Cas9 knockdown, short hairpin RNA-mediated knockdown, or viral transduction. TCR-responsive transcription factors, including MYC, IRF4, BATF, and NFATC1, are responsible for the initial expansion of activated T cells and drive metabolic switches (175, 176). Key factors such as BLIMP1, T-bet, ID2, and RUNX3 ensure the robust functionality of these cells (177). The development of memory T cells is promoted by TCF1, EOMES, ID3, BACH2, and BCL-6 (178–180). Furthermore, the organization of resident T cell function is regulated by BLIMP1, ZNF683 (also known as Hobbit), and RUNX3 (181, 182). In the long term, T-cell exhaustion, which is commonly observed in chronic infections and tumors, is driven by the TOX, IRF4, BATF, and NR4A family of transcriptional regulators (183).

#### 4.2.4 Modulation of epigenetic targets

Epigenetic changes mediated by loss of function of TET methylcytosine dioxygenase 2 (TET2) have been observed in CD19-targeted CAR-T cells in patients with chronic lymphocytic leukemia. These changes lead to altered differentiation and the formation of T_CM_-like cells with long-term persistence (184). Additionally, these patients exhibited higher levels of granzyme B in CAR-T cells compared to others, indicating improved effector function. In a mouse melanoma model, knockdown of TET2 in overtly metastatic OT-I cells resulted in delayed melanoma progression, with a reduction in tumor size of up to 80%. This knockdown also reduced T-cell exhaustion when compared to wild-type T cells (185). Similarly, in models of acute and chronic viral infection, knockdown of DNMT3A and SUV39H1 promoted the formation of memory precursor T cells and reduced the number of differentiated cells. This led to increased resistance to exhaustion and prolonged secretion of IL-2 and IFN-γ (186, 187). These findings suggest that knockdown of these genes maintains chromatin accessibility in CAR-T cells, promotes CAR-T cell persistence and stemness, and potentially enhances antitumor therapy by providing IL-2 and IFN-γ supplementation.

#### 4.2.5 Overcoming immunosuppressive solid TME

The tumor microenvironment (TME) affects CAR-T cell persistence and function through stromal cell networks, extracellular matrix proteins, and immune cells. These factors contribute to T-cell exhaustion and hinder their ability to persist. To overcome this, strategies are needed to increase the resistance of CAR-T cells to immunosuppressive mechanisms and maintain their antitumor activity. In the TME, glucose dysregulation affects T-cell mitochondrial function through lactate acidification, increases the expression of inhibitory checkpoint receptors, and suppresses T-cell function (188, 189). IL-7 improves T-cell survival by increasing glucose uptake through the facilitation of glucose transporter 1 transport (190). On the other hand, IL-15 decreases glycolytic enzyme expression by reducing m-TORC activity and enhances T-cell survival through mitochondrial biogenesis, promoting fatty acid oxidation. This leads to the formation of hypo-differentiated T cells with increased glucose uptake and mitochondrial activity, promoting catabolic oxidative phosphorylation (191, 192). In a study conducted on glycerol diacyl kinase (DGK)-deficient CAR-T cells, it was found that preventing dysregulation of DAG metabolism improved the antitumor function in mouse mesothelioma and glioblastoma xenograft models (193, 194). Additionally, CAR-T cells generated through the Notch signaling pathway, known as T_SCM_-like CAR-T cells, exhibited enhanced persistence, proliferative capacity, and improved antitumor efficacy mediated by downstream FOXM1 activity. This suggests that differences in metabolism and signaling pathways play a crucial role in determining the generation and antitumor efficacy of T_SCM_-like cells (195). Moreover, hypoxia, which negatively regulates the tumor microenvironment (TME) in von Hippel-Lindau disease tumor suppressor (VHL) deficiency and hypoxia-inducible factors, was found to sustain glycolysis in chronically stimulated T cells. This sustained glycolysis promotes the expression of cytotoxic molecules, such as granzyme B, and enhances tumor control (196).

#### 4.2.6 Immune checkpoints

Immune checkpoints play a crucial role in tumor therapy, showing significant results in the treatment of specific solid tumors (197). Inhibitory checkpoint receptors such as PD1, TIM3, CTLA4, and LAG3 are responsible for negatively regulating TCR signaling and are often utilized by TME cells that express a high level of ligands for these receptors (198) (**Table 1**). Currently, the CTLA4-specific antibody ipilimumab and the PD1-specific antibodies nivolumab, pembrolizumab, and cemiplimab have been approved for the clinical treatment of melanoma and certain lung cancers (197). In the case of solid tumors, combining anti-PD1 with bisulfite ganglioside (GD2)-targeted CAR-T cell therapy for neuroblastoma has shown some improvement in T cell expansion or persistence, leading to therapeutic effectiveness (199). Additionally, CRISPR-mediated knockdown of PDCD1 (which encodes PD1) in CAR-T cells has demonstrated enhanced CAR-T cell performance in both B cell and solid tumor models, resulting in improved *in vivo* anti-tumor responses (200, 201). In a mouse model of chronic infection, the deletion of PD1 leads to the eventual differentiation of depleted CD8^+^ T cells over the long term. This suggests a physiological role for PD1 in limiting T cell activation and depletion (202). While blocking and disrupting immune checkpoint receptors enhances the functional activity of CAR-T cell effectors, it does not promote the long-term persistence of CAR-T cells or control of solid tumors. Therefore, it is crucial to carefully consider the patient’s tumor type and optimize the approach when deciding whether targeting immune checkpoint receptors is appropriate for ACT (161).

**Table 1:** Treatment of immune checkpoints in different tumors.

References	Tumor types	Therapy of ICIs	Previous therapie	Study design
Arina V Zinkina-Orikhan et al. (2019)	melanoma	anti-PD-1 with or without anti-CTLA-4 treatment		117 participants (NCT03913923)
Pfizer CT. (2023)	melanoma	nivolumab or pembrolizumab and ipilimumab		150 participants (NCT05926960)
Nashat Gabrail et al. (2022)	hematological malignancies and lymphoma	CD47/SIRPα		72 participants (NCT05293912)
Peihua Luet et al (2021)	Advanced Solid Tumors	Anti-CAR-NK Cells		40 participants (NCT05194709)
Panayiotis Savvides, et al. (2020)	Non Small Cell Lung Cancer	PD-1/PD-L1/CTLA-4 + gene therapy		85 participants (NCT04495153)
Han weidong (2020)	Solid Tumor	TLR-7 agonists	PD-1 or CD47 antibody combination	50 participants (NCT04588324)
Marcia Hodik (2023)	Glioblastoma	mRNA-LP vaccines		28 participants (NCT04573140)
Els Wauters (2020)	Lung Diseases, InterstitialNSCLCImmunotherapy	PD-1/PD-L1/CTLA-4	chemotherapy	70 participants (NCT04807114)
ImmunityBio, Inc. (2018)	Melanoma; NSCLC; SCLC	PD-1/PD-L1 + IL-15		147participants (NCT03228667)
Michele Maio (2020)	MelanomaNon Small Cell Lung Cancer	PD-1 + CTLA-4	chemotherapy	184 participants (NCT04250246)
Hazel Buncab (2023)	Undifferentiated Pleomorphic SarcomaLiposarcomaSynovial SarcomaOsteosarcomaEwing Sarcoma	PD-1 + AXL	PD-1 inhibitor + CAB-AXL-ADC	120 participants (NCT03425279)

* Above data from clinicaltrials.gov/.

In this study, we investigated the role of antigen-presenting cells (APCs) in the tumor microenvironment (TME) and their impact on T-cell function. We found that a deficiency in APCs in the TME can result in T-cell functional failure (203). To address this issue, we explored the use of mRNA vaccines combined with single or multiple antigens. These vaccines effectively activate APCs, leading to the production of type I interferons and inflammatory factors. These factors are then cross-presented to CD8^+^ T cells, which helps restore their recognition ability and enhances the anti-tumor effect. Additionally, we examined the combination of type I interferons, inflammatory factors, adoptive T-cell therapy (CAR-T cells), and inhibitors of the PI3K-AKT pathway, WNT signaling activation, transcription factors regulation, modulation of epigenetic targets, overcoming immunosuppressive solid TME, and immune checkpoint regulation. These strategies can enhance adaptive immune responses either individually or in combination. Targeted interventions can be made based on the innate immune recognition between RNA sensing and DNA sensing mechanisms. For instance, cGAS/STING agonists can be used to produce type I IFN and inflammatory factors, which can enhance antigen-presenting cell (APC) function. This approach may also increase the number of central memory T cells (T_CM_ cells)and stem cell memory T cells(T_SCM_ cells) and delay terminal differentiation or CD8^+^ T recognition, thus facilitating the initiation of the adaptive immune response. Additionally, supplementation of type I-IFN can lead to macrophage polarization alteration, enhancing APC antigen recognition and presentation, and ultimately boosting the adaptive immune response and anti-tumor effects. Further research is needed to determine the applicability of these strategies in patients with different types of solid cancers and develop effective treatment approaches.

## 5 Conclusion

Tumors pose a significant threat to health, particularly glioblastoma’s ‘cold’ immunity. Although there has been progress in comprehending the innate immune response to tumors and viruses mediated by various DNA/RNA sensors, several key unknowns remain regarding the overlap between DNA and RNA sensing mechanisms. The interaction between DNA and RNA sensing mechanisms allows the host to move throughout the tumor and virus spectrum to eliminate invading pathogens and effectively prevent host damage. It is important to note that the viral and immune microenvironment employ multiple strategies to evade host innate immune responses against tumors and viruses by inhibiting this overlap. Progress in these fields will facilitate the creation of viral vaccines or adjuvants, as well as therapeutics that specifically target nucleic acid sensors. This will enhance traditional treatments and immunotherapies. This review focuses on the recognition mechanism between DNA and RNA sensing in the innate immune response. It also highlights how the generation of type I IFN promotes APC cross-starting of the adaptive immune response, providing a theoretical basis for tumor therapy.

## Author contributions

W-SL: Conceptualization, Writing – original draft, Writing – review & editing. Q-QZ: review– original draft. QL: Investigation, Writing & original draft. S-YL: Investigation, Writing & original draft. G-QY: Writing – review & editing. Y-WP: Writing – review & editing.
